# Orexin Depolarizes Central Amygdala Neurons via Orexin Receptor 1, Phospholipase C and Sodium-Calcium Exchanger and Modulates Conditioned Fear

**DOI:** 10.3389/fnins.2018.00934

**Published:** 2018-12-18

**Authors:** Erik T. Dustrude, Izabela F. Caliman, Cristian S. Bernabe, Stephanie D. Fitz, Laura A. Grafe, Seema Bhatnagar, Pascal Bonaventure, Philip L. Johnson, Andrei I. Molosh, Anantha Shekhar

**Affiliations:** ^1^Department of Psychiatry, Institute of Psychiatric Research, Indiana University School of Medicine, Indianapolis, IN, United States; ^2^Paul and Carole Stark Neurosciences Research Institute, Indiana University School of Medicine, Indianapolis, IN, United States; ^3^Department of Anatomy and Cell Biology, Indiana University School of Medicine, Indianapolis, IN, United States; ^4^Program in Medical Neuroscience, Indiana University School of Medicine, Indianapolis, IN, United States; ^5^Department Anesthesiology and Critical Care, Children’s Hospital of Philadelphia, University of Pennsylvania Perelman School of Medicine, Philadelphia, PA, United States; ^6^Janssen Research & Development, LLC, San Diego, CA, United States; ^7^Indiana Clinical and Translational Sciences Institute, Indiana University School of Medicine, Indianapolis, IN, United States

**Keywords:** orexin (hypocretin), orexin receptor 1 (OX1R), central amygdala, fear conditioning, chemogenetic

## Abstract

Orexins (OX), also known as hypocretins, are excitatory neuropeptides with well-described roles in regulation of wakefulness, arousal, energy homeostasis, and anxiety. An additional and recently recognized role of OX is modulation of fear responses. We studied the OX neurons of the perifornical hypothalamus (PeF) which send projections to the amygdala, a region critical in fear learning and fear expression. Within the amygdala, the highest density of OX-positive fibers was detected in the central nucleus (CeA). The specific mechanisms underlying OX neurotransmission within the CeA were explored utilizing rat brain slice electrophysiology, pharmacology, and chemogenetic stimulation. We show that OX induces postsynaptic depolarization of medial CeA neurons that is mediated by OX receptor 1 (OXR1) but not OX receptor 2 (OXR2). We further characterized the mechanism of CeA depolarization by OX as phospholipase C (PLC)- and sodium-calcium exchanger (NCX)- dependent. Selective chemogenetic stimulation of OX PeF fibers recapitulated OXR1 dependent depolarization of CeA neurons. We also observed that OXR1 activity modified presynaptic release of glutamate within the CeA. Finally, either systemic or intra-CeA perfusion of OXR1 antagonist reduced the expression of conditioned fear. Together, these data suggest the PeF-CeA orexinergic pathway can modulate conditioned fear through a signal transduction mechanism involving PLC and NCX activity and that selective OXR1 antagonism may be a putative treatment for fear-related disorders.

## Introduction

Deregulation of fear responses are thought to contribute to a number of anxiety disorders, including posttraumatic stress disorder, panic, and phobias associated with panic disorder (Milad et al., 2006; Herry et al., 2008; Hofmann, 2008; Holmes and Singewald, 2013). These disorders can have severe consequences on quality of life and are accompanied with serious economic burden for society (Olesen et al., 2012). There is an emerging role for the orexin (OX) system in the regulation of fear behaviors (Sears et al., 2013; Soya et al., 2013, 2017; Flores et al., 2014, 2017).

Orexins (OX) were simultaneously discovered by two research groups and are also known as hypocretins (de Lecea et al., 1998; Sakurai et al., 1998). The OX system consists of two neuropeptides, orexin A (OX A/hypocretin 1) and orexin B (OX B/hypocretin 2), derived from a common prepro-OX precursor, which act as endogenous ligands for G-protein-coupled receptors OXR1 and OXR2 (HcrtR1/2). OX-producing neurons are highly concentrated within the hypothalamus (PeF), a region demonstrated to produce anxiety and panic in rodents (Shekhar and DiMicco, 1987) and in humans (Shapira et al., 2006). Multiple previous studies have implicated the OX system in modulating fear responses: intracerebroventricular injection of OXR1 antagonists enhances fear extinction in rats (Flores et al., 2014); OXR1 knockout mice display impaired freezing responses and reduced amygdala neuron activity (Soya et al., 2013); dual OX antagonist almorexant reduces fear-potentiated startle response (Steiner et al., 2012); immobility time after footshock positively correlates with prepro-OX mRNA expression and is attenuated by treatment with a dual OX antagonist (Chen et al., 2014); and increased OX neuron activity is correlated to higher freezing levels and resistant fear extinction (Sharko et al., 2016).

OX-producing neurons have robust projections to numerous brain regions implicated in arousal and emotional responses including the locus coeruleus (LC), dorsal raphe nucleus, bed nucleus of the stria terminalis, periaqueductal gray, and the amygdala (Peyron et al., 1998; Trivedi et al., 1998; Marcus et al., 2001). Amygdala nuclei, including lateral (LA), basal (BLA), and central (CeA), are involved in the formation and expression of conditioned fear responses and are therefore potential sites for OX-modulation of fear (Fanselow and LeDoux, 1999; LeDoux, 2000; Davis and Whalen, 2001; Walker and Davis, 2002). In a majority of terminal fields, OX exerts its effects via postsynaptic excitation. This postsynaptic effect is mediated by distinct intracellular signaling machinery that vary between brain regions (Yang and Ferguson, 2002, 2003; Yang et al., 2003; Kolaj et al., 2007; Doroshenko and Renaud, 2009; Chen et al., 2017). Preliminary studies of OX signaling within the CeA suggested that the neuropeptides induce postsynaptic excitation (Bisetti et al., 2006), but the receptor selectivity and signal transduction mechanism is unknown and presynaptic modulation has not been explored.

Here, using OX receptor subtype-specific antagonists, C 56, a selective OXR1 antagonist (Bonaventure et al., 2015) and JNJ10397049, a selective OXR2 antagonist (Dugovic et al., 2009), and prepro-OX-DREADD (Grafe et al., 2017), we aimed to characterize the cellular, molecular, and behavioral consequences of OX signaling in the CeA.

## Materials and Methods

### Animals

Experiments were performed using 150–200 g male Sprague-Dawley rats (Harlan/Envigo, Indianapolis, IN, **RRID**:RGD_1566457). Rats were group housed in plastic cages in standard housing conditions (maintained at 22°C) for a minimum of 4 days prior to experimental procedures with *ad libitum* access to food and water and 12:12 light/dark cycle (lights on at 07:00 h). All experiments were conducted in accordance with the Guide for the Care and Use of Laboratory Animals (Institute for Laboratory Animal Research, The National Academies Press) and the guidelines of the IUPUI Institutional Animal Care and Use Committee.

### Stereotaxic Surgery

To allow adequate viral expression, male (40–50 g) Sprague-Dawley rats were utilized for stereotaxic injection of AAV virus 4 weeks prior to electrophysiology experiments. An AAV containing the Gq-coupled Designer Receptor Exclusively Activated by Designer Drugs (DREADD) construct (Armbruster et al., 2007) under control of the prepro-OX gene promoter (Ple112) (Moriguchi et al., 2002) was produced by the Penn Vector Core and obtained from Dr. Seema Bhatnagar (AAV1-Ple112-hM3Dq(Gq)-mCitrine). Rats were anesthetized under isoflurane delivered via nose cone (2–3%/vol MGX research Medicine, Vetamac, Rossville, IN, United States, in medical air, Praxair). During anesthesia, a warming pad was used to maintain core body temperature and corneal and paw-withdrawal reflexes were monitored to insure adequate anesthesia level. The animals head was shaved and a 10 mm incision was made to expose the skull. Within a stereotaxic apparatus for rodents (900 series Ultraprecise Kopf Instruments, Tujunga, CA, United States), lambda and bregma were set to equivalent dorsal-ventral positions. For DREADD expression, 800 nl bilateral injections were performed in 40–50 g rats targeting the PeF (15° angle toward midline AP: -1.9; ML: 2.8; DV -7.6). For drug delivery during behavior experiments, intra-CeA infusions were performed via two stainless steel guide cannulae (26 gauge, Plastics One, Roanoke, VA, United States) implanted bilaterally in 150–175 g rats targeting the CeA (AP: -2.4 ML: 3.8 DV: -7.8). The guide cannulae were secured using three 2.4 mm screws anchored to the skull with cranioplastic cement. Dummy cannulae (Plastics One, Roanoke, VA, United States) with lengths matching the guide cannulae were placed inside the guide cannulae to prevent occlusions until treatment. Rats were sutured following completion of surgery and administered 0.025 mg/kg buprenorphine 4 times at 12 h intervals to mitigate pain. DREADD-expressing animals were returned to their home cages for 4 weeks to permit high viral expression at the time of experiments and cannulae-implanted animals were returned to their home cages for 1 week to permit recovery. These surgery weights and study designs allowed for weight matching at the time of data collection (150–200 g).

### Electrophysiology

Rats (150–200 g) were anesthetized with isoflurane, as described above, transcardially perfused with 60 ml ice-cold oxygenated NMDG solution (30 ml/min) and immediately decapitated. (NMDG solution in mM: 92 NMDG; 2.5 KCl; 1.25 NaH_2_PO_4_; 10 MgSO_4_; 0.5 CaCl_2_; 30 glucose; 30 NaHCO_3_; 5 Na-ascorbate; 3 Na-pyruvate; 20 HEPES; 2 Thiourea, 315 mOsm, 7.4 pH). Brains were then rapidly removed, placed in ice-cold oxygenated NMDG solution and coronal slices (350 μM) were prepared containing the amygdala. Slices were incubated at 31°C for 12 min in oxygenated NMDG solution before being placed in room temperature oxygenated artificial cerebrospinal fluid (ACSF) until recording. (ACSF solution in mM: 130 NaCl; 3.5 KCl; 1.1 KH_2_PO_4_; 1.3 MgCl_2_; 2.5 CaCl_2_; 30 NaHCO_3_; 10 glucose, 315 mOsm, 7.4 pH). Cells were identified for recording at 40× magnification using Scientifica Slicescope microscope under DIC illumination (Scientifica, Uckfield, United Kingdom). ACSF was warmed to 30°C and perfused at a rate of 2–3 ml/min during recordings. Compounds were added to ACSF at desired concentrations (clozapine-N-oxide, CNO; 1 μM; tetrodotoxin, TTX; picrotoxin 50 μM; 1 μM; C 56: 1 μM; JNJ10397049; 1 μM; KB-R7943; 80 μM; NiCl: 3 mM; U73122 10 μM). Whole-cell patch-clamp recordings were obtained using standard techniques with a Multiclamp 700B amplifier, pClamp 10.3 software, and a Digidata 1440 interface (Molecular Devices, Sunnyvale, CA, United States). Borosilicate glass electrodes (WPI, Sarasota, FL, United States) (resistance 3–6 MΩ) were prepared with a potassium gluconate based recording solution (Internal solution in mM: 140 K-gluconate; 2 KCl; 3 MgCl_2_; 10 HEPES; 5 Phosphocreatine; 2 K-ATP; 0.2 Na-GTP, 290 mOsm, 7.4 pH). Cells were excluded if greater than 10% change to series resistance was observed during the experiment. Holding potential was adjusted to -60 mV at the beginning of agonist/antagonist experiments and 10 min baseline was established before application of compounds. To verify sensitivity to OX, the neuropeptide was perfused for a minimum of 3 min which we determined to prohibit wash-out effect. Experiments utilizing antagonists included an additional 10 min baseline period of antagonist perfusion prior to perfusion of OX neuropeptides and antagonists were maintained in the solution during OX treatment. Compound 56 (C 56) was provided by Janssen Research & Development, all other compounds were purchased from Tocris (Bio-Techne, Minneapolis, MN, United States), and salts used in patching solutions were purchased from Sigma Aldrich (St. Louis, MO, United States). A membrane holding potential of -60 mV was chosen (i) to allow direct comparisons between cells and conditions, (ii) because this is very near the resting membrane potential of CeA neurons, and (iii) because OX-mediated depolarization to action potential threshold was uncommon from -60 mV using our recording solutions. CeA neuron firing in response to OX can be achieved by solutions that shift excitatory balance or by holding neurons at more depolarized potentials and these differences make direct comparisons between studies very difficult (Bisetti et al., 2006; Johnson et al., 2012). Negative current injection was utilized to hyperpolarize the cell membrane before and during orexin application to monitor changes to input resistance. Presynaptic glutamate release was examined by evoking paired post synaptic potentials (EPSPs) from a holding potential of -70 mV with a 120 ms inter-potential interval. A concentric, platinum/iridium, bipolar electrode (FHC, Bowdoin, ME, United States) and Master8 pulse stimulator (A.M.P.I., Jerusalem, Israel) were utilized to provide stimulation. The electrode was placed medial to the CeA and five recordings separated by 10 s were averaged for each data point. The peak amplitude of the second pulse was divided by the peak amplitude of the first pulse to generate the paired pulse ratio (PPR) value. A hyperpolarizing pulse immediately preceded stimulation so that input resistance could be assessed over the course of the experiment. Orexin did not alter input resistance thus allowing current clamp recordings to be used to estimate synaptic paired-pulse responses. Application of compounds during synaptic release experiments was counterbalanced in our design by randomizing the order of conditions applied to individual cells. Each condition was applied by perfusion for at least 8 min prior to recording.

### Modified Solutions

In recordings where potassium driving force was altered, modified ACSF was utilized wherein KCl was adjusted from 3.5 to 14 mM. At 30°C this shifted the equilibrium potential from -97 to -60 mV resulting in net zero potassium conductance at -60 mV recording potential. In recordings where sodium driving force was altered, NaCl was replaced with NMDG-Cl, NaHCO_3_ was reduced to 15 mM, and 2 mM KCl in the internal solution was replaced by 3 mM NaCl. These changes resulted in a shift of equilibrium potential from +175 to +40 mV. In recordings where calcium driving force was altered, normal ACSF containing 100 μM BAPTA-AM was utilized to chelate intracellular calcium. For this series of experiments, picrotoxin 50 μM was included to remove GABA-A current contributions that result from altered reversal potentials. Equilibrium potentials for individual ions were predicted using the Nernst Equation.

### Immunohistochemistry

Four weeks after PeF-targeted prepro-OX DREADD virus injection, rats were anesthetized with isoflurane and transcardially perfused with 250 ml 0.1 M phosphate buffered saline (PBS) and then 250 ml 0.1 M sodium phosphate buffer (PB) containing 4% paraformaldehyde. Brains were removed and postfixed for 12 h before 2× rinsing in PB and placement in 0.1 M PB containing 30% sucrose for 48–72 h. Serial coronal sections (30 μm) were cut using a Leica freezing microtome (Buffalo Grove, IL, United States) and immediately placed in cryoprotectant solution (30% ethylene glycol and 30% glycerol in 0.1 M PB). Prior to immunohistochemical processing, slices were stored at –20°C. Then, free-floating sections were washed in 0.1 M PBS for 30 min before incubation in 1% H_2_O_2_ in PBS for 20 min. Next, sections were washed in PBS for 30 min and in PBS with 0.3% Triton X-100 (PBST) for 10 min. Following washes, an overnight incubation (approximately 12–16 h) in PBST was performed at room temperature with either rabbit anti-OXA-polyclonal (cat. no. H-003-30, Phoenix Pharmaceuticals, Burlingame, CA, **RRID**:AB_2315019; diluted 1:18,000) or mouse anti-green fluorescent protein (GFP, cat. no. A11120, Molecular Probes, Eugene, OR, United States **RRID**:AB_221568; diluted 1:1,000) antibodies against OX or mCitrine, respectively. The next day, sections were washed for 30 min in PBS and incubated with either horseradish peroxidase (HRP) goat anti-rabbit (for anti-OXA) or HRP horse anti-mouse (for anti-GFP) IgG antibodies for 90 min (cat nos. PI-1000/**RRID**:AB_2336198 and PI-2000/**RRID**:AB_2336177, respectively, Vector Laboratories, Burlingame, CA, United States; diluted 1:200). Following secondary antibody incubation, slices were washed in PBS for 30 min and then underwent a 10 min tyramide signal amplification according to the manufacturer’s protocol (cat no. NEL700A001KT, PerkinElmer, Waltham, MA, United States; diluted 1:250). Following an additional 30 min wash in PBS, sections were incubated with either Cy3- (for anti-OX A) or Alexa488-conjugated streptavidin (for anti-GFP) for 30 min (cat nos. 016-160-084/**RRID**:AB_2337244 and 016-540-084/**RRID**:AB_2337249, respectively, Jackson ImmunoResearch, West Grove, PA, United States; diluted 1:200). After a final 30 min wash in PBS, sections were cover slipped with mounting media (cat. no. H-1200, Vector, Burlingame, CA, **RRID**:AB_2336790). Images were obtained using a Nikon A1R+ scanning microscope (Melville, NY, United States). Identical microscope, capture settings and linear LUTs were used within the amygdala. PeF images were taken at reduced laser power due to increased intensities of fluorescence of orexinergic cell bodies compared to fibers. Quantification of fibers was performed by drawing ROI of amygdala regions according to Paxinos and Watson’s rat brain atlas (Paxinos and Watson, 2005) and recording the average intensity in arbitrary fluorescent units (A.U.) for each ROI with Nikon NIS Elements software.

### Intracerebral Injections

To infuse OXR1 antagonist compound 56 to the CeA, internal cannulae that extend 1.0 mm beyond the guide cannulae (Plastics One, Roanoke, VA, United States) were inserted and connected via polyethylene tubing to 10 μl microsyringes (Hamilton, Reno, NV, United States). An injection of 300 pmol/100 nl was delivered using a Harvard PHD 2000 (Harvard Apparatus, Inc., South Natick, MA, United States) syringe pump at a rate of 100 nl/min. Cannulae remained inserted for 1 min after infusion to allow for diffusion. Formulation of vehicle was 4.6% 1N HCl, 4.6% 1N NaOH, and 90.8% 30% (w/v) SBE-β-Cyclodextrin. Animals were excluded when cannulae placements were not within the CeA or amygdala region (BLA/LA) within both hemispheres (based on coronal brain sections, Paxinos and Watson atlas), when extensive damage was observed at the injection site, and when enlarged ventricles were observed. Formulation was maintained for injections that were administered at 10 mg/kg.

### Fear Conditioning

The day prior to conditioning, rats were handled and habituated to the conditioning box (25.5 × 25.5 × 39.5 cm) for 10 min. The conditioning box was situated in a larger sound-attenuated chamber (Ugo Basile, Monvale, Italy), which was illuminated with a white 15-Lux light. A speaker in the rear wall of the chamber was operating during all sessions to provide white noise. The floor of the conditioning box was constructed of parallel stainless-steel bars and connected to a scrambled shock generator (Ugo Basile, Monvale, Italy). Before each trial, the chamber and the conditioning box were cleaned with 70% ethanol to remove olfactory cues. On the acquisition day, rats were treated 30 min prior to fear training with 30 mg/kg JNJ10397049, or either 10 mg/kg, 30 mg/kg i.p., or 300 pmoles/100 nl cannula injection of compound 56. Five trials consisted of a 20 s, 4 kHz, 80 dB tone that co-terminated with a 0.5 s, 0.8 mA foot shock [inter-trial interval (ITI) 60 s]. This data was analyzed by comparing the first and last 20 s bins that correspond to the tone. Rats were allowed to explore the chamber for 100 s before conditioning began and remained in the chamber for 60 s after the last trial. The following day, the same rats were subjected to a second treatment of orexin antagonist or vehicle and tested for conditioned fear responses (freezing). In this test, rats were exposed to eight conditioned stimulus (CS) tones (4 kHz, 80 dB, 20 s, ITI 60 s). Total time freezing during the CS presentations (20 s bin) were recorded and scored for each rat, and this number was expressed as a percentage of the total CS. Freezing was defined as the absence of all movement except for normal breathing. Animals that did not acquire fear (less than 10% freezing on acquisition day) were excluded.

### Statistical Analysis

All data are represented as means ± SEM. The *n*-value for each group is given in figure legends. Electrophysiological data was recorded as response per cell and averaged for each condition. For all experiments, a single condition contains a minimum of three animals to aid reproducibility. All analyses were performed using GraphPad Prism 6 (Prism 6, GraphPad Software, La Jolla, CA, United States). Two-way ANOVA with Sidak’s *post hoc* test was used for multiple comparisons and one-way ANOVA with Sidak’s *post hoc* test was used where appropriate. In the results, reported statistics are for two-way ANOVA unless otherwise specified. Symbols denote statistical significance meeting *p* < 0.05 threshold. Datasets are available upon request.

## Results

### Orexinergic Projections Mediate Depolarization of CeA Neurons

**FIGURE 1:**
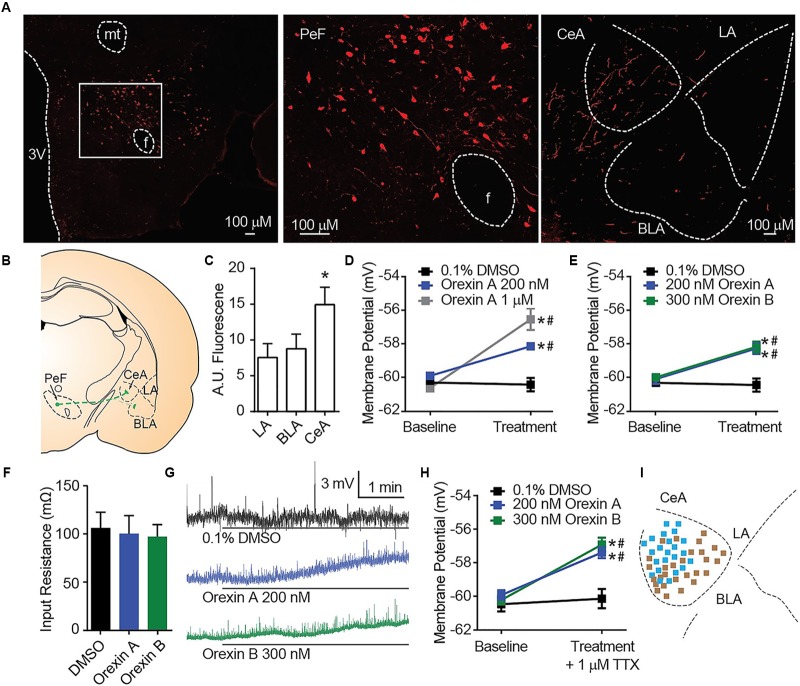
OX mediates postsynaptic depolarization of CeA neurons. **(A)** Representative 20× images revealed OX A positive cell bodies in the PeF (f is fornix, -2.80 mm bregma) and OX A positive fibers in the amygdala (-2.40 mm bregma). Boxed area is enlarged to show individual OX A-positive PeF neurons. **(B)** Schematic of PeF projections to the amygdala and **(C)** summary quantification of fluorescence, *n* = 7 animals. **(D)** Depolarization response to OX A was observed for CeA neurons (*n* = 9–15 cells). **(E)** CeA neurons depolarized to applications of either OX A or OX B (*n* = 10–12 cells). **(F)** Input resistance of CeA neurons before and during OX perfusion. **(G)** Representative traces of OX mediated depolarization. Solid bars under traces indicate perfusion of OX or vehicle. **(H)** Depolarization persisted following 1 μM TTX treatment indicating a postsynaptic effect (*n* = 9–11 cells). **(I)** Mapping of OX sensitive (blue, 23/51 recordings) and insensitive (brown) neurons in the CeA shows a pattern of sensitivity in the medial CeA. Data are means ± SEM, symbols indicate significance by Sidak’s *post hoc* test, *p* < 0.05, ^∗^ between subjects, # within subjects.

Orexin A immunostaining reveals amygdala nuclei innervation by orexinergic fibers (Figures 1A,B). In alignment with a previous report that qualitatively observed dense OX innervation of the CeA versus the LA or BLA (Peyron et al., 1998), we detected ∼70% more fluorescence in the CeA than other amygdala nuclei [one-way ANOVA region effect *F*_(2,__12)_ = 14.16, *p* = 0.0007, Sidak’s *p* ≤ 0.0036 for CeA versus LA or BLA] (Figure 1C). To examine the cellular consequences of OX innervation of the CeA, we applied OX neuropeptides to acute brain slices. Here, compared to time control vehicle group, we detected depolarization of CeA neurons in response to bath application of OX A [interaction *F*_(2,__33)_ = 15.58, *p* < 0.0001, time effect *F*_(1,__33)_ = 45.42, *p* < 0.0001]. Application of 200 nM OX A significantly depolarized CeA neurons compared to vehicle and 1 μM OX A significantly depolarized CeA neurons compared to 200 nM OX A and vehicle conditions (Sidak’s between subjects *p* ≤ 0.0003, within subjects *p* ≤ 0.0008) (Figure 1D). We additionally observed that CeA depolarization was produced by application of either OX neuropeptide OX A or OX B [interaction *F*_(2,__24)_ = 10.25, *p* < 0.0006, time effect *F*_(1,__24)_ = 42.81, *p* < 0.0001]. Application of 200 nM OX A or 300 nM OX B significantly depolarized CeA neurons compared to vehicle (Sidak’s between subjects *p* ≤ 0.0002, within subjects *p* < 0.0001) without affecting input resistance (Figures 1E–G). To determine the dependence of depolarization upon presynaptic or postsynaptic machinery, we examined response to OXs in the presence of 1 μM tetrodotoxin (TTX). TTX is a voltage-gated sodium channel blocker that isolates postsynaptic effects by blocking presynaptic activity. Significant OX-mediated depolarization was maintained in these experiments [interaction *F*_(2,__58)_ = 6.877, *p* = 0.0021, time effect *F*_(1,__58)_ = 36.32, *p* < 0.0001, Sidak’s between subjects *p* < 0.0001, within subjects *p* < 0.0001] suggesting a requirement of postsynaptic OX receptors expressed on the recorded CeA neuron (Figure 1H).

In a previous report, the depolarizing effect of OX on CeA neurons was observed for medial CeA neurons but was not examined in lateral CeA neurons (Bisetti et al., 2006). As the major output of the amygdala to autonomic and hypothalamic regions, activity of the medial CeA could be predictive of behavioral phenotypes (Bisetti et al., 2006). Here, we chose to record from cells throughout the entire CeA and observed a pattern of OX sensitive neurons (blue, 23 of 51) in the medial CeA and OX insensitive neurons (brown 28 of 51) in the lateral CeA (Figure 1I).

### Orexin Mediated Depolarization of Central Amygdala Neurons Occurs via OXR1 and Not OXR2

**FIGURE 2:**
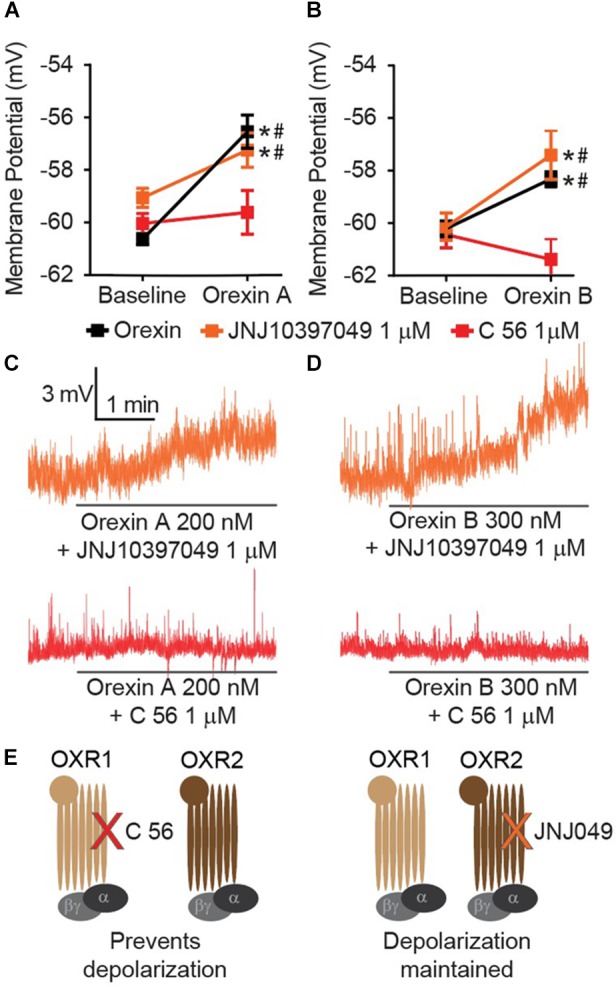
CeA depolarization to OX occurs via OXR1 but not OXR2. **(A)** Summary data for the application of OX A, or **(B)** OX B, in the absence or presence of receptor antagonists (OXR1 antagonist C 56 in red, and OXR2 antagonist JNJ10397049 in orange, *n* = 9–13 cells per condition). **(C)** Representative traces of OX A conditions and **(D)** OX B conditions. Solid bars under traces indicate perfusion of OX following 10 min perfusion of antagonist. **(E)** Schematics illustrating that OXR1 antagonism prevents OX mediated depolarization and OXR2 antagonism does not affect OX mediated depolarization. Data are means ± SEM, symbols indicate significance by Sidak’s *post hoc* test, *p* < 0.05, ^∗^ between subjects, # within subjects.

Orexin release contributes an excitatory input to CeA neurons (Bisetti et al., 2006), but the mechanism by which this occurs has not been completely described. Orexin A and B are produced from a common prepro-OX precursor protein and are ligands for two G-protein coupled receptors, OXR1 and OXR2. OXR2 displays equivalent affinity for each OX A and OX B, whereas OXR1 displays greater affinity for OX A than OX B (Sakurai et al., 1998). We have observed that either OX A or OX B can produce depolarization of CeA neurons (Figure 1G). Recently developed selective OXR1 and OXR2 antagonists allow for comprehensive experiments to delineate OX-mediated cellular mechanisms. We examined CeA depolarization, mediated by OX A and OX B in the presence of selective OXR1 antagonist C 56 (1 μM) and selective OXR2 antagonist JNJ10397049 (1 μM) (Figures 2A–D). Bath application of either OX A or OX B induced significant depolarization of CeA neurons [OX A interaction *F*_(2,__26)_ = 7.234, *p* = 0.0032, time effect *F*_(1,__26)_ = 30.58, *p* < 0.0001; OX B interaction *F*_(2,__24)_ = 15.61, *p* < 0.0001, time effect *F*_(1,__24)_ = 20.09, *p* = 0.0002] (Figures 2A,B). In response to OX treatment following OXR2 antagonism, CeA neurons were significantly depolarized (Sidak’s between subjects *p* ≤ 0.0002, within subjects *p* < 0.0001). However, OXR1 antagonism with C 56 prevented OX A or OX B-induced CeA neuron depolarization (red symbols, Sidak’s between subjects *p* ≥ 0.07374, within subjects ≥ 0.1685). These data suggest OX-mediated depolarization of CeA neurons is primarily via activation of OXR1 and not via OXR2 (Figure 2E).

### Chemogenetically Mediated OX Transmission Induces CeA Neuron Depolarization

Having observed depolarization of CeA neurons following bath application of OX neuropeptides (Figure 1), we hypothesized that stimulating activity of orexinergic PeF neurons would recapitulate this response by inducing synaptic transmission of OXs within the CeA. To specifically activate this subgroup of CeA projecting fibers, we utilized a chemogenetic DREADD (Armbruster et al., 2007) under control of the prepro-OX gene promoter (Moriguchi et al., 2002). This virus has been previously validated to express DREADD-coupled G_αq_ in OX neurons and stimulate release of OX in response to DREADD agonist clozapine-N-oxide (CNO) (Grafe et al., 2017).

**FIGURE 3:**
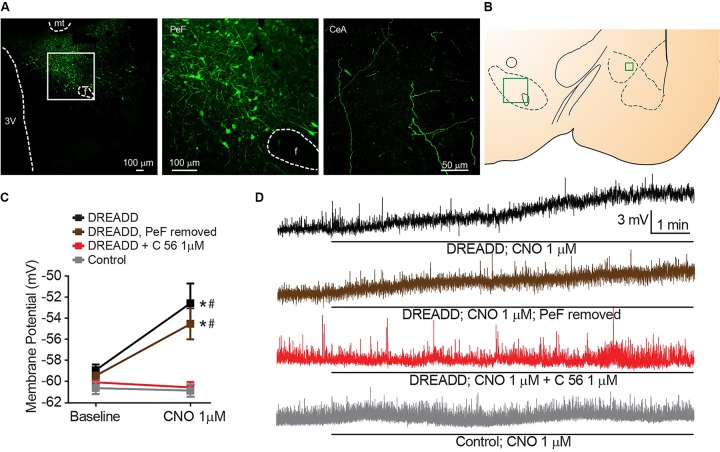
Stimulated release of endogenous OX produces OXR1 dependent CeA neuron depolarization. **(A)** Representative PeF (20×) and CeA (40×) images demonstrate DREADD-mCitrine-positive cell bodies and fibers of cells expressing prepro-OX-DREADD-mCitrine virus. Boxed area is enlarged to show individual mCitrine-positive PeF neurons. **(B)** Schematic of regions that were imaged (-2.40 mm bregma for CeA, -2.80 mm bregma for PeF). **(C)** Summary data of Clozapine-N-oxide (CNO), DREADD agonist, effects for the indicated conditions (*n* = 9–13). **(D)** Representative traces from CNO treated neurons. CNO stimulated depolarization in animals expressing PeF-targeted DREADD virus except when OXR1 was antagonized by C 56. Solid bars under traces indicate perfusion of CNO. Data are means ± SEM, symbols indicate significance by Sidak’s *post hoc* test, *p* < 0.05, ^∗^ between subjects, # within subjects.

We targeted expression of DREADD selectively in the PeF OX neurons by stereotaxic surgery and guide cannula positioned, bilaterally, within the PeF to infect cell bodies (Figures 3A,B). Fibers of OX-producing neurons that originate in the PeF were activated in acute brain slices by 1 μM CNO DREADD agonist and significant CeA neuron depolarization was observed [interaction *F*_(3,__29)_ = 4.607, *p* = 0.0094, time effect *F*_(1,__29)_ = 9.066, *p* = 0.0054]. This depolarization confirms functional connectivity between orexinergic PeF and CeA neurons (black symbols, Sidak’s between subjects *p* ≤ 0.0010, within subjects *p* = 0.0002) (Figures 3C,D). We hypothesized that CeA depolarization was due to local release of OX from PeF terminal fields within the CeA. To test this, we removed the medial section of brain slices to excise the PeF and DREADD-expressing orexinergic cell bodies. Here, we again observed significant CeA neuron depolarization following 1 μM CNO treatment (brown symbols, Sidak’s between subjects *p* ≤ 0.0200, within subjects *p* = 0.0431) confirming that modulation of CeA depolarization by DREADD-stimulated release of OX is due to local CeA terminal fields. To test whether DREADD-stimulated synaptic transmission from orexinergic neurons and subsequent CeA neuron depolarization is dependent upon OXR1, we antagonized the receptor with 1 μM C 56 prior to CNO application. OXR1 antagonism prevented DREADD-mediated depolarization thereby recapitulating the requirement of OXR1 activity for OX-dependent CeA depolarization (Sidak’s between subjects *p* ≥ 0.9999, within subjects *p* ≥ 0.9992). An application of 1 μM CNO had no effect on CeA neurons from naïve animals (Sidak’s between subjects *p* ≥ 0.9999, within subjects *p* ≥ 0.9935) (Figure 3C).

### Orexin-Mediated Depolarization of CeA Neurons Requires Phospholipase C-Mediated Activity of Sodium-Calcium Exchanger

**FIGURE 4:**
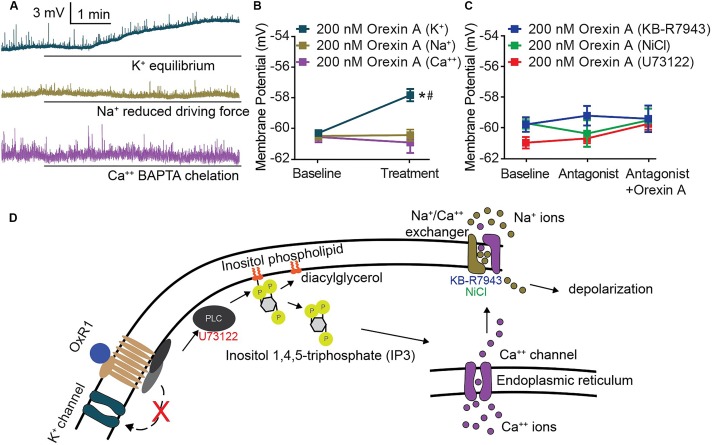
CeA depolarization to OX occurs via sodium and calcium conductance mediated by PLC and NCX activity. **(A)** Representative traces of CeA neurons and **(B)** summary data for OX A treated cells with altered ionic gradients. Solid bars under traces indicate perfusion of OX. CeA depolarization did not occur when sodium or calcium conductance was reduced, but was maintained when potassium conductance was eliminated (*n* = 8–11 cells). **(C)** Summary data for OX and antagonist treated cells. PLC antagonist 10 μM U73122 and NCX antagonists 80 μM KB-R7943 and 3 mM NiCl prevented OX mediated CeA depolarization (*n* = 7–10 cells). **(D)** Schematic of putative signaling pathway by which OX mediates postsynaptic depolarization via PLC and NCX without contribution to depolarization by potassium channels. Data are means ± SEM, symbols indicate significance by Sidak’s *post hoc* test, *p* < 0.05, ^∗^ between subjects, # within subjects.

Underlying the activity of all excitable membranes are choreographed shifts in conductance of specific ions (Hodgkin and Katz, 1949). Therefore, the requirement of an ion for OX-mediated CeA membrane depolarization can be determined by limiting or eliminating its conductance. We applied this strategy and utilized modified solutions to eliminate potassium conductance at resting membrane potential, reduce sodium equilibrium potential via ionic substitution, or chemically chelate internal calcium. Under each of these conditions, OX A-mediated depolarization of CeA neurons was examined. A significant depolarization following OX A treatment was maintained when potassium conductance was eliminated [interaction *F*_(2,__27)_ = 15.30, *p* < 0.0001, time effect *F*_(1,__27)_ = 9.831, *p* = 0.0041, Sidak’s within subjects *p* < 0.0001] and completely prevented when either sodium or calcium conductance was restricted (Sidak’s within subjects *p* ≥ 0.8050) (Figures 4A,B). Potassium conductance is the most commonly described effector of OX-mediated depolarization within the brain (Ivanov and Aston-Jones, 2000; Brown et al., 2001; Yang and Ferguson, 2003; Yang et al., 2003; Hoang et al., 2004; Murai and Akaike, 2005; Kolaj et al., 2007, 2008; Doroshenko and Renaud, 2009). However, potassium does not appear to regulate OX-mediated CeA depolarization compared to sodium and calcium, conductances of which are both required.

Previous studies have detailed OX responses in other brain regions as insensitive to pertussis toxin (Hoang et al., 2004) and sensitive to phospholipase C (PLC) inhibition (Yang et al., 2003). This indicates that OXs signal through G_q/11_ G-protein coupled receptors. Having observed dependence of OX-mediated depolarization upon both sodium and calcium conductance (Figure 4B), we hypothesized that CeA OX signaling requires sodium-calcium exchanger (NCX), a membrane protein that contributes to conductance of both ions. We examined contributions of PLC and NCX via antagonism of PLC by 10 μM U73122 and antagonism of NCX by 3 mM NiCl and by 80 μM KB-R7943. In each condition, application of the antagonist prevented significant depolarizing responses in response to OX A application, and the antagonists themselves had no effect on membrane potential [interaction *F*_(4,__28)_ = 1.078, *p* = 0.3862, time effect *F*_(2,__28)_ = 1.88, *p* = 0.3198] (Figure 4C). Based on these data, we put forward a putative cell signaling model for OXR1 activation in the CeA includes PLC-dependent calcium release and downstream NCX-mediated depolarization (Figure 4D).

### OX Modifies Presynaptic Glutamate Release Into the CeA via OXR1

**FIGURE 5:**
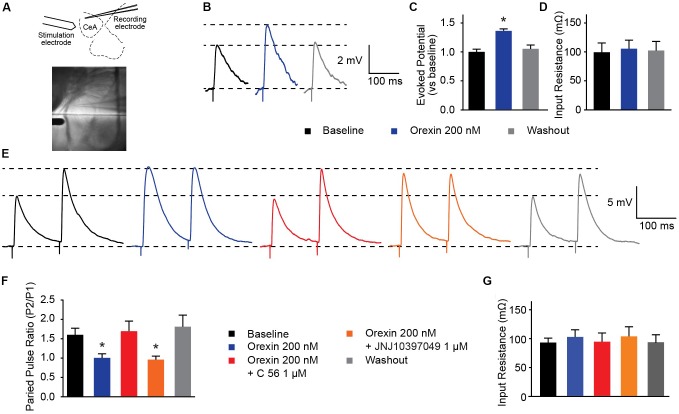
OXR1 modifies presynaptic glutamate release in the CeA. **(A)** Schematic and image depicting location of stimulation and recording electrodes during evoked excitation experiments. **(B)** Representative potential traces evoked from CeA neurons. **(C)** Summary data of potentials evoked from CeA neurons from a holding potential of -70 mV and **(D)** their recorded input resistance (*n* = 11). **(E)** Representative paired-pulse potential traces for indicated treatments. **(F)** Summary data of paired pulse ratios from CeA neurons treated with 200 nM OX A in the presence and absence of OXR antagonists (C 56 OXR1 antagonist and JNJ10397049 OXR2 antagonist) and **(G)** their recorded input resistance. Treatments were applied to the same cells in randomized orders (*n* = 5). Data are means ± SEM, symbols indicate significance by Sidak’s *post hoc* test, *p* < 0.05, ^∗^ between subjects.

Several groups have shown that OX neurons colocalize with, and release, the excitatory neurotransmitter glutamate (Torrealba et al., 2003; Henny et al., 2010; Schone et al., 2014). In the prefrontal cortex, presynaptic glutamate release can be regulated by OX (Aracri et al., 2015). To examine whether OX exerts effects on glutamate release in the CeA, we evoked excitatory postsynaptic potentials (eEPSPs) in combination with OX and OXR antagonists (Figure 5A). First, we observed that compared to baseline evoked potentials, 200 nM OX A increased excitatory potentials without modifying input resistance [one-way ANOVA treatment effect *F*_(2,__20)_ = 20.63, *p* < 0.0001]. This indicates that OX potentiates glutamate response (Figures 5B–D). Next we examined the PPR of two stimulations. PPR is sensitive to altered presynaptic calcium signaling and the availability of presynaptic vesicles (Regehr, 2012) such that a shift in the ratio is indicative of modifications to presynaptic machinery. We observed that 200 nM OX A shifted the PPR of CeA neurons by roughly 60% and that this shift requires activity of OXR1. Antagonism of OXR2 with 1 μM JNJ10397049 had no effect on PPR compared to OX A by itself, however, 1 μM OXR1 antagonist C 56 produced PPR comparable to baseline [one-way ANOVA treatment effect *F*_(4,__16)_ = 9.063, *p* = 0.0005]. The effects of these conditions were counterbalanced by applying treatments to recorded cells in random order and none of the conditions altered input resistance (Figures 5E–G). These data highlight that OX action through OXR1 alters CeA presynaptic glutamate release in addition to effects on postsynaptic membrane potential.

### OXR1 Antagonism in the CeA Reduces Expression of Conditioned Fear

It has been previously shown that intracerebroventricular delivery of OX enhances fear expression and that delivery of OX antagonists reduces fear expression (Flores et al., 2014). We have demonstrated that CeA depolarization and control of synaptic transmission is dependent upon OXR1 but not OXR2 activity, so we aimed to determine the effects of OXR antagonism on fear expression. Compound 56 and JNJ10397049 were utilized to antagonize OXR1 and OXR2 activity, respectively, 30 min prior to auditory fear conditioning and 30 min prior to fear expression testing. JNJ10397049 was administered I.P. at 30 mg/kg and compound 56 was administered either I.P. at 10 or 30 mg/kg or bilaterally via cannulae targeting the CeA at 300 pmoles/100 nl at both time points.

**FIGURE 6:**
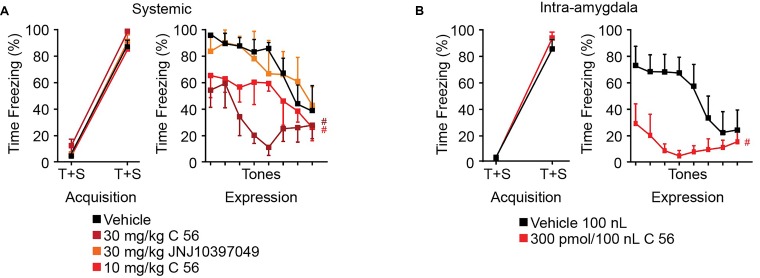
OXR1 antagonism reduces expression of cued fear in rodents. **(A)** Summary data of auditory fear conditioning and expression following systemic I.P. injections of vehicle, C 56 at 10 and 30 mg/kg, and JNJ10397049 at 30 mg/kg. **(B)** Summary data of auditory fear conditioning and expression following cannulae injection of 100 nl vehicle or 300 pmole C 56. None of the treatment conditions affected fear acquisition. OXR1 antagonism, but not OXR2 antagonism, reduced expression of cued fear. Data are means ± SEM, symbols indicate significance by Fisher’s LSD *post hoc* test, *p* < 0.05, # treatment effect.

In both systemic and intra-amygdala experiments, vehicle, C 56, and JNJ10397049 treated animals acquired fear, observed as time effect and measured as difference in time freezing during the first and fifth tone [systemic time effect *F*_(1,__19)_ = 328.9, *p* < 0.0001; intra-amygdala time effect *F*_(1,__9)_ = 354.8, *p* < 0.0001] (Figures 6A,B). On the following day, freezing response during 8 consecutive tones was evaluated in the absence of a paired shock. Here, we observed significant tone and treatment effects suggesting that animals began to extinguish fear over time and that OX antagonism affected freezing [two-way ANOVA systemic tone effect *F*_(7,__91)_ = 6.789, *p* < 0.0001, treatment effect *F*_(3,__13)_ = 6.194, *p* = 0.0076]. Animals treated with JNJ10397049 displayed similar freezing responses to vehicle treated animals (black versus orange, Fisher’s LSD *post hoc* analysis, *p* = 0.8624) suggesting that OXR2 antagonism did not modify fear expression. Alternatively, both 10 and 30 mg/kg treatments with C 56 reduced fear expression compared to vehicle (bright and dark red, Fisher’s LSD *post hoc* analysis, *p* = 0.0477 and *p* = 0.0018) suggesting that OXR1 activity is required for normal fear expression (Figure 6A).

We next administered C 56 via cannulae targeted to the CeA (Figure 6B and Supplementary Figure 1). This allowed us to examine the requirement of CeA OX signaling for fear expression. Here, C 56 treated animals demonstrated significantly reduced freezing responses [two-way ANOVA intra-amygdala tone effect, *F*_(7,__70)_ = 2.824, *p* = 0.0119, treatment effect, *F*_(1,__10)_ = 12.85, *p* = 0.0050] (Figure 6B). Therefore, OXR1-mediated signaling contributes to CeA-dependent expression of fear without affecting acquisition of fear memories.

## Discussion

Our report advances the understanding of OX signaling mechanisms that modulate the activity of CeA neurons and influence fear behavior. A previous report demonstrated that intracerebroventricular injection of an OXR1 antagonist enhances the extinction of fear memory (Flores et al., 2014). Moreover, OXR1 knockout promotes extinction of conditioned fear (Soya et al., 2013). These findings align with our own data demonstrating reduced fear expression following systemic and intra-CeA antagonism of OXR1. There is evidence that in addition to OXR1 activity in the CeA, OXR1 activity in the locus coeruleus (LC) is critical for fear learning. Through the interruption of OX projections to the LC, consolidation of fear acquisition is impaired (Sears et al., 2013). Furthermore, OXR1-null mice display enhanced extinction of fear memory that is reversed by virus-mediated recovery of OXR1 expression exclusively within LC neurons (Soya et al., 2013, 2017). Thus, a key role of OXR1 in regulating fear behavior has been established for both the CeA and LC. The Sears et al. study also put forward that there is no direct role for OXR1 signaling in the lateral amygdala for control of fear memories. Our data shows their finding may be the result of amygdala-level organization of OX-positive terminal fields wherein orexin fiber innervation of the CeA is 100% greater than the lateral amygdala. We have demonstrated that intra-CeA infusion of an OXR1 antagonist prior to fear conditioning and again prior to fear expression prevents expression of fear. This correlates with our *in vitro* electrophysiology experiments showing OXR1 modulated depolarization of the medial CeA. Together, our data has validated the CeA as an important region for OX modulation of fear memories. With our dual-treatment approach prior to acquisition and prior to expression, reduced freezing could result from either preventing expression of fear during expression testing or preventing consolidation following acquisition. Future experiments are poised to determine the detailed role of CeA OXR1 in acquisition and/or consolidation of fear memories.

OX has emerged as an excitatory neuropeptide that engages diverse signaling cascades downstream of the postsynaptic receptor. OX-mediated excitation has been described to require various combinations of activity by PLC, protein kinase C, nonselective cation channels, altered sodium, calcium or potassium conductance, and NCX (Eriksson et al., 2001; Yang and Ferguson, 2002, 2003; Yang et al., 2003; Kolaj et al., 2007; Doroshenko and Renaud, 2009; Chen et al., 2017). The predominantly described mechanism of depolarization is reduction of potassium conductance which facilitates slow and moderate depolarization of neurons in several brain regions (Ivanov and Aston-Jones, 2000; Brown et al., 2001; Yang and Ferguson, 2003; Yang et al., 2003; Hoang et al., 2004; Murai and Akaike, 2005; Kolaj et al., 2007, 2008; Doroshenko and Renaud, 2009). Gq-linked receptors, such as OXRs, can modulate conductance of various potassium channels including GIRK and TASK (Cui et al., 2010; Wilke et al., 2014). A previous study of CeA OX signaling demonstrated an OX mediated shift of potassium ramp current (Bisetti et al., 2006). However, when we eliminated contribution of potassium conductance, CeA neurons depolarized in response to OX treatment (Figure 4). We demonstrate that OX-mediated depolarization of CeA neurons require NCX activity and resemble what is reported for histaminergic neurons of the tuberomammillary nucleus without a requirement for potassium conductance (Eriksson et al., 2001).

In the present study, we mapped the OX-sensitive and OX-insensitive neurons within the CeA. We show a pattern of OX sensitivity within the medial CeA and OX insensitivity within the lateral CeA (Figure 1H). Within current models of CeA circuitry (Badrinarayan et al., 2012), medial CeA neurons output pro-fear behavioral responses whereas lateral CeA neurons contribute to either direct GABAergic inhibition or disinhibition of the CeM. The pattern of OX sensitivity in the CeM that we have observed would permit OX mediated pro-fear signaling to occur. Lack of OX-sensitive neurons in the CeL is not surprising due to the dual role of the region in positive and negative control of the CeM during cured fear behaviors (Badrinarayan et al., 2012). Our observed pattern of OX-sensitivity as well as the postsynaptic dependence of OX mediated CeA depolarization is consistent with a report by Bisetti et al. ((2006). Bisetti reported that in response to equivalent applications of OX A and OX B, CeM neurons produced depolarization responses with similar amplitudes (Bisetti et al., 2006). Since OXR2 is expected to demonstrate equal receptor occupancy for both OX A and OX B neuropeptides (Sakurai et al., 1998), the authors hypothesized that OX effects in the CeA may be mediated by OXR2. However, agonist occupancy of OX GPCRs might not be predictive of receptor selectivity. Our study has been assisted by the development of OXR1 and OXR2 specific antagonists (Dugovic et al., 2009; Bonaventure et al., 2015). We pharmacologically isolated OX mediated effects on CeA neurons and made the novel observation that CeA depolarization by OX requires OXR1, but not OXR2 (Figure 2). These findings were confirmed using an AAV-OX-DREADD virus to drive synaptic release of OX in the CeA. The current study utilized this virus to identify connectivity of OX-PeF neurons and the CeA and molecular requirements of OXR1 signaling in the CeA. However, additional detailed behavior studies using this virus during examinations of animal fear behavior would help us to further understand the role of OX in fear memories formation. An additional novel finding of our study is that OX modifies presynaptic glutamate release in the CeA, again via OXR1 but not OXR2. This observation is similar to the presynaptic effect of OX in the prefrontal cortex (Aracri et al., 2015). These mechanistic findings demonstrating OXR1-mediated effects in the CeA are recapitulated by animal behavior experiments that demonstrate OXR1 contribution to fear expression.

OXR2 does not appear to influence the effects of OX in the CeA, but the receptor has emerged as critical signaling machinery for mediating wakefulness (Mang et al., 2012). This function of OX has recently been targeted for treatment of insomnia with suvorexant, a dual antagonist of OXR1 and OXR2 (Dohme, 2013; Jacobson et al., 2014; Patel et al., 2015). Alternatively, OXR1 has been implicated in hypothalamic-pituitary-adrenal response to repeated stress and in expression of fear behavior (Heydendael et al., 2011; Soya et al., 2013, 2017). Separate modalities for OXR1 versus OXR2 could allow for specific targeting of OXR1 function to relieve fear-related symptoms.

## Author Contributions

ED, AM, and AS contributed to the conception and design of the study. ED, IC, and AM preformed electrophysiological experiments. CB and SF preformed animal surgeries and behavior experiments. ED and IC preformed immunohistochemistry and confocal analysis. LG and SB provided the DREADD virus and technical assistance for its use. PB provided compound 56 and technical assistance for its use. PJ and SF analyzed animal behavior experiments. ED wrote the first draft of the manuscript. IC wrote sections of the manuscript. All authors contributed to manuscript revision, read, and approved the submitted version.

## Conflict of Interest Statement

The authors declare that the research was conducted in the absence of any commercial or financial relationships that could be construed as a potential conflict of interest.
