# Supplementary material for: Orexin Depolarizes Central Amygdala Neurons via Orexin Receptor 1, Phospholipase C and Sodium-Calcium Exchanger and Modulates Conditioned Fear
Source: Front Neurosci. 2018 Dec 18;12:934. doi: 10.3389/fnins.2018.00934 (PMC6305451; doi:10.3389/fnins.2018.00934)
Supplement: Supplementary file 1 [file Data_Sheet_1.docx]

Supplementary Material

**Orexin Depolarizes Central Amygdala Neurons via Orexin Receptor 1, Phospholipase C and Sodium-Calcium Exchanger and Modulates Conditioned Fear**

Erik T. Dustrude, Izabela F. Caliman, Cristian S. Bernabe, Stephanie D. Fitz, Laura A. Grafe, Seema Bhatnagar, Pascal Bonaventure, Philip L. Johnson, Andrei I. Molosh^*^, Anantha Shekhar^*^

*** Correspondence:** Dr. Anantha Shekhar, ashekhar@iu.edu; Dr. Andrei I. Molosh, amolosh@iu.edu


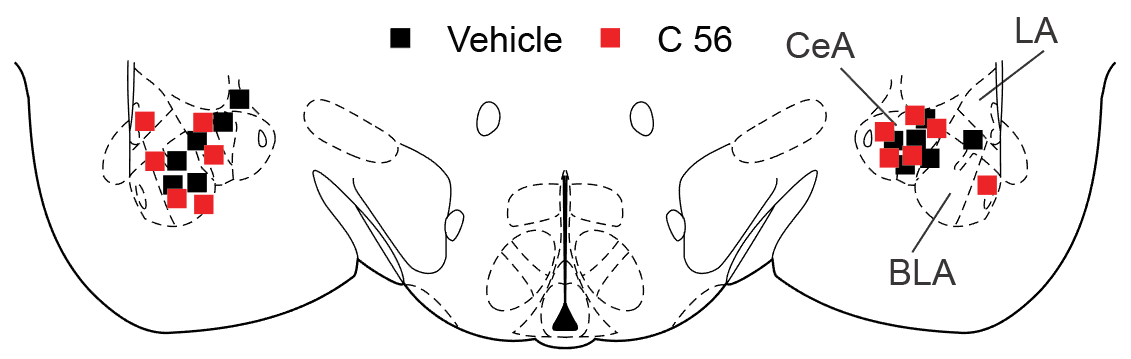


**Supplementary Figure 1.** CeA targeted cannulae placement. The illustration depicts a coronal brain section containing the amygdala, -2.40 mm from bregma. Black boxes represent cannulae placement for vehicle experiments and red boxes represent cannulae placement for C 56 antagonism experiments.
